# MicroRNAs and ovarian function

**DOI:** 10.1186/1757-2215-5-8

**Published:** 2012-02-09

**Authors:** Jason Baley, Julang Li

**Affiliations:** 1 Department of Animal and Poultry Science, University of Guelph, Guelph, Ontario, Canada

**Keywords:** microRNA, gene regulation, ovaries, small RNA

## Abstract

MicroRNAs (miRNAs) are a class of small non-coding RNAs which function in gene regulation with an important role in cell proliferation, maturation, and activity. The regulatory role of these small RNA molecules has recently begun to be explored in ovarian cells, uncovering their influence on gonadal development, steroidogenesis, apoptosis, ovulation, and corpus luteum development. This emerging area of research has extended and reshaped our understanding on how ovarian function is regulated. Here, we review the current understanding of miRNA biogenesis, the role and mechanism that miRNAs play in post-transcriptional gene expression regulation, and specifically the current evidence of miRNA involvement in ovarian development and function. Future comprehensive understanding of the role of miRNAs in the ovary in both physiological and pathological conditions may offer new treatment strategies for infertility and other ovarian disorders.

## 

MicroRNAs (miRNAs) are small (19-25 bp) RNAs that diversely regulate gene expression through their decrease of messenger RNA (mRNA) stability or translation [1-3]. The functions of these non-coding RNAs, until recently, have been relatively unknown, and are emerging as important regulators controlling diverse physiological and pathological processes including cell division, differentiation, migration and apoptosis [2,3]. Ovarian development involves proliferation and differentiation of germ cells and somatic cells. The correct completion of these processes is dependent on the coordinated expression of genes in a spatially and temporally specific manner. Consequently, gene expression is highly regulated and controlled at both the transcriptional and translational level. It is thus conceivable that miRNAs also play an important role in ovarian development. Here we review some of the recent findings on the potential roles of miRNA in ovarian functions.

### MicroRNA biosynthesis, function and degradation

The genes that encode miRNAs, which comprise a class of naturally occurring, small non-coding RNAs, are generally transcribed by RNA polymerase II, processed into short hairpin RNAs by the enzyme Drosha and its RNA-binding cofactor DiGeorge syndrome critical region gene 8 (DGCR8), as shown in Figure 1[4-7]. Within the nucleus, these two proteins convert primary miRNA (pri-miRNA) to ~70-100 base precursor-miRNA (pre-miRNA) that contain a characteristic hairpin loop. The pre-miRNAs are exported from the nucleus to the cytoplasm, and further processed by another enzyme, Dicer (encoded by Dicer1), giving rise to mature miRNAs. These are then transferred to Argonaute proteins, members of the argonaute (Ago) protein family, in an Argonaute-containing RNA Induced Silencing Complex (RISC) and elicit their effects by binding within the 3'-untranslated region (3' UTR) of target mRNAs [8]. There are some special cases in which the miRNA do not undergo the regular processing steps during biosynthesis. Mirtrons are a subset of miRNAs which utilize an alternative pathway for miRNA biogenesis [9]. These miRNAs are located within short introns, and once splicing is complete, a debranching enzyme generates the pre-miRNA-like hairpin which can then be exported from the nucleus to the cytoplasm. Generally, mirtrons compose only a small percentage of genomically encoded miRNAs, as the sequences of mirtrons are not evolutionarily conserved [10]. It was proposed that the conversion of a short intron into a mirtron may represent an evolutionary opportunistic strategy for the development of new gene regulating RNAs [11]. Once the mature miRNA duplex is produced, it usually loses one of its strands (the complementary strand*) as the presence of structure imperfections within this duplex facilitates the disposal of the complementary strand from the RISC Loading Complex (RLC). The mature miRNA strand is then loaded onto the Argonaute-containing RISC (miRISC) which facilitates gene silencing [12]. Recognition is thought to mainly involve base pairing of miRNA nucleotides 2-8, representing the seed sequence [13]. The miRISC then uses the miRNA strand as a guide to search for mRNAs in which the 3'UTR is complementary to the miRISC seed sequence. It has been estimated that 30-90% of messenger RNAs may be subjected to miRNA regulation, and individual miRNAs are predicted to target up to several hundred genes [14-16]. Many highly regulated mRNAs contain multiple miRNA binding sites, often targeted by different miRNAs, which may enhance the effectiveness of regulation [17]. MiRNAs exert their effect by negatively regulating gene expression through one of two mechanisms, i.e., mRNA degradation or translational suppression. The mechanism by which the miRNA proceeds is dependent on the complementarity between the miRNA and its mRNA target [18-20]. A high level of complementarity corresponds to the degradation of the target mRNA via the RNA-mediated interference (RNAi) pathway. When there is inadequate complementarity, miRNAs bind to the 3'UTR of the target mRNAs and translational suppression occurs through miRISC. Plants more commonly use the first mechanism while animals more often utilize the second [2]. It was proposed that there is a competition between miRISC and eIF4E for association with the mRNA 5' cap structure. eIF4E is a eukaryotic translation initiation factor which binds to the 7-methyl guanosine cap structure present at the 5' UTR of cellular mRNA, consequently delivering it to the eIF4E translation initiation complex [21]. MiRNA can repress translation by interfering with the ability of the 7-methyl guanosine cap structure at the 5' end of mRNA to engage the translation initiation complex, which is normally mediated by eIF4E [22]. Evidence of the competition between miRISC and eIF4E is supported by experiments in which the eIF4E translation initiation complex is artificially tethered to mRNA, resulting in resistance of translational repression by miRNAs [22]. This cap-dependent mechanism of translation inhibition is also supported by experiments indicating that some mRNAs lacking the 7-methyl guanosine cap structure are not able to be repressed by miRNAs [22]. If miRISC does in fact compete with eIF4E then it would be predicted that providing excess eIF4E would alleviate repression. This is in fact the case when purified eIF4E is added to the system [23]. Ago2 proteins play a role in miRNA regulation of gene expression as the Mid domain of Ago2 has been proposed to resemble eIF4E, with two phenylalanine residues in the Mid sequence adopting equivalent positions to the eIF4E tryptophans. Mutation of the phenylalanines impairs the ability of Ago2 to repress translation [24]. In addition, miRNAs may also increase translation of specific mRNAs in a manner dependent on the cell cycle [25], and a large number of miRNAs may be expressed in a tissue-specific manner [26].

**Figure 1:**
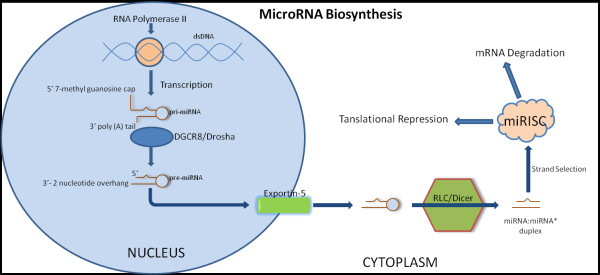
**Model of microRNA (miRNA) biogensis/transportation/function**. In the nucleus, miRNA are transcribed by RNA polymerase II into single strand RNA, which folds into a double stranded primary miRNA (pri-miRNA). Pri-miRNA are cleaved by Drosha yielding the precursor miRNA (pre-miRNA). Pre-miRNA are then translocated from the nucleus via exportin 5 to the cytoplasm. Once in the cytoplasm pre-miRNA are further cleaved by the RNase III endonuclease Dicer into a double stranded miRNA. Only one strand of the duplex is stably associated with the miRISC complex. The miRNA strand is usually favoured more than the miRISC* strand, although there are exceptions. The double stranded miRNA then separates into two single strands, one strand is then degraded and the other is incorporated into a complex with argonaute proteins, forming the RNA induced silencing complex (RISC). RISC then binds to target mRNA causing translational repression or degradation. Adapted and modified from [7].

### Small RNA in the ovary

The ovary performs numerous roles critical for oocyte development and ovulation. Within the ovary, granulosa cells are an important site for estrogen production for local use as well as providing endocrine signalling to other tissues [27]. The somatic cells of the ovary act to support the growth and development of the oocyte in preparation for the surge of luteinizing hormone (LH) which elicits a physiological response leading to ovulation. This response includes promoting meiosis, steroidogenesis, follicular development, cumulus cell expansion, luteinization, and progesterone production, ultimately promoting oocyte maturation [28,29].

The role of small RNA in the ovary is indicated by the fact that knockout of Dicer, the ribonuclease III which processes pre-small RNA to mature functional small RNA in the ovary resulted in the dysfunction of folliculogenesis, oocyte maturation, ovulation and infertility [30-34]. Piwi interacting RNAs (piRNAs), miRNA, and small interfering RNA (siRNA) are the major small RNAs present in the ovary. PiRNA is primarily expressed and functions in the germ cell [35]. Endogenous siRNA utilize the well-known RNA interference pathway to regulate gene expression [36,37]. In this regulatory system, endogenous double stranded RNAs (dsRNAs) are thought to derive from pseudogenes that encode a complementary mRNA allowing for formation of dsRNA templates, Dicer cleavage of the dsRNA then generates endogenous siRNAs [36,37]. With respect to endogenous siRNAs, they have been identified to play a role in oocytes [36,37]. Knockout of DGCR8, which should specifically block the miRNA pathway, and leave the endogenous siRNA pathway intact helps to distinguish the effects of these closely related classes of RNA species (i.e., miRNA and siRNA) [38]. Targeted deletion of oocyte DGCR8, an enzyme specific for miRNA biosynthesis, had no significant adverse effect on maturation of oocytes [39], indicating that the role of miRNA synthesized via this pathway in the oocyte is limited. However, miRNA could also be generated by direct transcription of short hairpin RNAs and mirtrons (i.e., pre-miRNA that arise from intron excision) which short circuit the standard miRNA biogenesis process by eliminating the need for Drosha/DGCR8 mediated cleavage [11,40]. It is thus still unknown if miRNAs generated via these pathways play a role in oocyte growth and maturation. Nevertheless, increasing evidence support the notion that influence of miRNA on ovarian function primarily occurs through their actions on ovarian somatic cells such as granulosa cells. In order to determine the possible involvement of miRNAs in reproductive tissues, expression levels have been measured indicating the presence of large numbers of miRNA within the ovary. However, the functional importance of individual miRNA and the identity of their mRNA targets are now beginning to be discovered.

### MicroRNA in gonadal and early embryo development

Fetal gonadal development involves coordinated expression of thousands of genes, and it is conceivable that miRNAs are involved in the regulation of these genes during fetal development. In a recent study by Torley et al. (2011), key genes involved in fetal ovarian development were examined as potential targets of miRNA regulation [41]. This study provides evidence that miR-22 is up-regulated during testicular development and down-regulated during fetal ovarian development, correlating to increased estrogen receptor 1 (ESR1) expression levels during fetal ovarian development. This study suggests there is a repressive effect by miR-22 on estrogen signalling as a result of its influence on ESR1, indicating Sertoli cell development requires suppression of estrogen during testicular development [41].

Bannister et al.(2009), has shown that miRNAs are expressed in a sexually dimorphic manner in embryonic chicken gonads during sexual differentiation [42]. To further test the hypothesis that up-regulation of microRNA 202* (miR-202*) is specifically associated with testicular differentiation, estrogen modulation was used to feminize male (ZZ) gonads and masculinize female (ZW) gonads in order to study the association between male-biased miR-202* expression and gonadal sex differentiation. In ovo injection of estradiol-17 beta resulted in feminization of male gonads which was accompanied by a reduction in miR-202* expression levels, similar to the female phenotype. When estrogen synthesis was blocked, female gonads were masculinized, which is associated with increased miR-202* and down-regulation of the ovary specific Forkhead box L2 (FOXL2) gene, and up-regulation of testis associated genes; double sex and mab3 related transcription factor 1 (DMRT1) and SRY (sex determining region Y) box 9 (SOX9) genes. These results suggest that miR-202* may be involved in regulating gonadal development [43].

Newborn ovary homebox gene (NOBOX) is a transcription factor and NOBOX mRNA and protein are expressed in oocytes throughout folliculogenesis [44]. The importance of this gene is evident as NOBOX knockout mice are infertile, and mutations in the NOBOX gene have been associated with premature ovarian failure (POF), suggesting the essential role of NOBOX in folliculogenesis. As a potential miRNA recognition element (MRE) for miR-196a was identified in the 3'UTR of bovine NOBOX mRNA, a previous study investigated the possible regulatory effects of miR-196a on NOBOX expression [44]. During early embryogenesis, miR-196a steadily increases while NOBOX gradually decreases. This inverse relationship supports the proposed role of miR-196a as a regulator of NOBOX during early embryogenesis. To confirm the mechanism of this regulation, HeLa cell transfection studies and Western blot assays were conducted in which a significant inhibition of NOBOX expression was observed in HeLa cells expressing both NOBOX and miR-196a, in comparison to cells transfected with NOBOX alone. To determine the ability of miR-196a to regulate endogenous NOBOX expression, bovine early embryos were microinjected with miR-196a which resulted in reduced NOBOX mRNA and protein levels compared to negative control embryos, suggesting miRNA not only inhibit translation, but may also induce clearance of target mRNA [44].

Recent studies have also illustrated the importance of maternally inherited miRNAs in the oocyte for early embryonic development. One such maternal effect gene is Nucleoplasm 2 (NPM2), which is an oocyte specific nuclear protein essential for maternal and paternal DNA remodelling during early embryonic development, and is positively associated with oocyte developmental competence [45]. An inverse correlation was reported between miR-181a and NPM2 during early embryogenesis. Co-expression studies with NPM2 and miR-181a indicated a reduced expression of NPM2 protein in miR-181a-expressing cells compared to control cells without miR-181a, suggesting that translation of NPM2 is repressed by miR-181a and a key role of this miRNA in regulating early embryonic development [45].

### Hormonal regulation of microRNA expression in the ovary

Recent evidence supports the role of luteinizing hormone (LH)/human chorionic gonadotropin (hCG) in the regulation of miRNA expression [46]. Ovarian granulosa cells are likely an important target for LH-regulated, miRNA-mediated changes in gene expression during LH-induced luteinization. Following LH/hCG stimulation in the ovarian cells, miR-132 and miR-212 which share the same seed sequence were found to be highly up-regulated, suggesting their potential role in ovarian response to LH. It is suggested that miR-132 and miR-212 play an important role as post-transcriptional regulators in granulosa cells, as computational analysis has identified 77 putative mRNA as potential targets of miR-212, and miR-132 in granulosa cells [46]. C-terminal binding protein 1 (CTBP1) is a known target of miR-132, and the gene product acts as a co-repressor of nuclear receptor genes. Interestingly, knockdown of both miR-212 and miR-132 resulted in decreased protein levels of CTBP1 but with no change in mRNA levels [46]. Further studies to determine how these miRNAs cause these changes in CTBP1 expression would be useful to establish the precise relationship. These results suggest that miRNA may play a key role in tuning the gene expression cascade to allow for ovulation and the differentiation of luteal cells.

Follicle Stimulating Hormone (FSH) controls the development of granulosa cells during folliculogenesis by stimulating their proliferation and differentiation [47]. In particular, FSH regulates expression of several granulosa cell genes during FSH induced steroidogenesis. Examples of these FSH regulated genes include insulin like growth factor binding protein 3 (IGFBP3), steroidogenic acute regulatory protein (StAR), as well as numerous bone morphogenetic proteins (BMPs) [48-51]. The primary trigger of immature preantral follicle development into preovulatory follicles is FSH. A study reported that after 12 hours of FSH exposure, 17 miRNAs were up regulated and 14 miRNAs were down regulated. In this same study progesterone levels in the medium were up regulated by FSH at 12 hours [47], suggesting that miRNAs may play a role mediating changes in gene expression and thus hormone production in granulosa cells following FSH exposure. Future molecular analysis would be important for confirming the specific binding between miRNA and the 3'UTR of potential target genes to better understand the role of miRNA in the phenotypic changes occurring following FSH exposure. Furthermore, the association between the proposed miRNAs and potential target genes regulating progesterone production are to be verified. The same study also revealed a bi-phase regulation of miRNAs by FSH. A significant decrease in the expression levels of miR-29a and miR-30d during the first 12 hours post FSH exposure was observed [47]. However, FSH induced a 2 and 3 fold increase in the expression of miR-29a and miR-30d at 48 hours post-FSH exposure, respectively [47]. This finding suggests that these two miRNAs could be involved in the fine tuning of FSH mediated granulosa cell function. In addition, it was also demonstrated that granulosa cells collected immediately before and 4 h after the ovulatory surge of LH/hCG exhibit differential miRNA expression patterns [46], suggesting a role in ovulation. Interestingly, a lack of miR17-5p and let7b, resulted in corpus luteum insufficiency and infertility in mice, and the phenotype was partially reversed by injection of miR17-5p and let7b into the ovaries in the mice [32], indicating the role of these miRNAs in corpus luteum formation.

### MicroRNA control of human ovarian cell steroidogenesis

Using a large-scale platform approach, it was recently shown that 51 microRNAs have suppressive effects on estradiol production [52]. MiRNAs shown to suppress the release of progesterone, androgens, and estrogens include miR-108, miR-135, miR-146, miR-19a, miR-20, miR-27, miR-28, miR-29, miR-125b, miR-126, miR-137, miR-184, miR-31, miR-105, miR-128, miR-129, miR-132, miR-140, and miR-188. It was hypothesized that these miRNAs act as physiological suppressors of general secretory activity [52]. In addition, over-expression of miR-24, miR-25, miR-122, miR-182, miR-18, miR-125, and miR-32 resulted in a rise in progesterone release, consistent with the process of luteinization [52]. Further study identifying the target genes of these miRNA in ovarian cells is critical in confirming the physiological function of these miRNAs. In another study, miR-224 was identified as one of 16 transforming growth factor- β1 (TGF-β1) regulated miRNAs in cultured murine granulosa cells, regulating granulosa cell proliferation via targeting Smad family member 4 (Smad4) in the TGF-β1 signal transduction pathway [53].

Our group has recently provided evidence that miR-378 is expressed in granulosa cells in an inverse manner compared to the expression of aromatase [54]. *In vitro *miR-378 over-expression and knockdown experiments revealed that aromatase expression, and therefore estradiol production, by granulosa cells, is post-transcriptionally down-regulated by miR-378. Furthermore, site-directed mutation studies identified two binding sites in the 3' untranslated region (UTR) of the aromatase coding sequence that are critical for the action of miR-378. We also provided further support for the mechanism of action by over-expressing the aromatase 3'UTR alone which resulted in the binding of miR-378 to the recombinant 3'UTR decreasing the opportunity for miR-378 to bind to the endogenous aromatase 3'UTR, reversing the repressive effect of miR-378 on aromatase protein levels [54].

### MicroRNA regulation of apoptosis in granulosa cells

LH induces ovulation and luteinization via its action on granulosa cells. During the LH induced transition of granulosa cells to luteal cells, apoptosis must be inhibited to allow for proper formation of a functional corpus luteum. A previous study has reported 13 miRNAs which are differentially expressed in murine granulosa cells before and 4 h after the hCG/LH surge, with miR-132, miR-212, and miR-21 being the top three highly up-regulated [46]. Further study by the same group revealed that when miR-21 expression was decreased to one twenty-seventh of its basal expression with locked nucleic acid (LNA-21) oligonucleotide transfection, apoptosis was induced in granulosa cells [55]. Similar results were observed in their in vivo study with a miR-21 inhibitor, although the targets of miR-21 suppressing apoptosis in granulosa cells are still to be identified [55].

In addition, Ma et al. (2011) investigated the expression of miR-378 and the interferon gamma receptor 1 (IFNGR1) gene at different luteal stages in bovine non-regressed and regressed corpus luteum using real time RT-PCR and Western blots analysis [56]. IFNGR1 plays a role in luteal cell apoptosis and was predicted to be a target of miR-378 [56]. Real time RT-PCR revealed that miR-378 expression was stage-dependent, with the highest level being detected in the late stages and the lowest level of expression occurring in the early stage of corpus luteum (CL) development. Western blot analysis of IFNGR1 revealed an inverse relationship between miR-378 and IFNGR1 protein. This data suggests that miR-378 may function in suppressing luteal cell apoptosis through the IFNGR1 gene [56]. It would be of interest to confirm the regulatory role of miR-378 with a gain and loss of function study.

### Ovarian pathologies and miRNAs as potential biomarkers

Recent studies have identified miRNAs as potential tissue specific biomarkers of disease and may provide a useful measure in the diagnosis of numerous pathologies [57]. Iorio et al. (2007), showed that in comparison to normal ovaries, numerous miRNA showed differential expression in epithelial ovarian cancer tissue [58]. It was found in ovarian cancer tissue that the most significantly over-expressed miRNAs include miR-200a, miR-141, miR-200c, and miR-200b. The most down-regulated miRNAs are miR-199a, miR-140, miR-145, and miR-125b1 [58]. However, the potential application of using differential expression of miRNA as a biomarker for disease would require a tissue biopsy.

In more recent studies, the relationship between circulating miRNAs and cancer has been investigated as exosomal miRNA have been shown to mirror the miRNA expression profile of the originating tumor cells [59]. These circulating tumor derived exosomes offer a potential non-invasive approach, as they do not require tissue biopsy for the detection of cancer. In a recent study by Zhou et al. (2011), the relationship between circulating miRNAs and premature ovarian failure (POF), an ovarian endocrine disorder which can have very serious implications for reproduction and overall health status, was investigated [60]. By comparing the miRNA expression profiles from the serum of POF and normal women, it was reported that ten miRNAs were up-regulated and two miRNAs were down-regulated in POF patients [60]. The miRNAs with increased expression were miR-202, miR-146a, miR-125b-2*, miR-139-3p, miR-654-5p, miR-27a, miR-765, miR-23a, miR-342-3p and miR-126, and the miRNAs with decreased expression were let-7c and miR-144 [60].

### Prospective

MicroRNAs are important regulators of cell fate determination, differentiation, proliferation, and tissue remodelling during development. Through complementary approaches, increasing evidence supports the important role of miRNAs in proliferation, apoptosis, and steroidogenesis within the ovary. However, knowledge of the underlying networks is still largely unclear. The biology of miRNAs in the ovary is a research area that is just starting to be explored. A greater understanding of these regulatory effects at the molecular level will be the next key step, and may allow for the potential usefulness of miRNAs and their inhibitors in control of human fertility, and reproductive disorders. Future studies to identify the important target genes as well as the use of animal models to monitor the phenotypic effects may allow for a more thorough understanding of the role played by miRNA in ovarian physiology. In addition, studies identifying the unique expression signature of miRNAs in certain ovarian pathological conditions such as polycystic ovarian syndrome, and ovarian cancer might offer an additional diagnostic tool to assess these disorders, and eventually provide insight for novel treatments of these diseases.

## List of Abbreviations

miRNA: microRNA; DGCR8: Digeorge syndrome critical region 8; pri: primary; pre: precursor; Ago: argonaute; RISC: RNA induced silencing complex; UTR: untranslated region; RLC: RISC loading complex; miRISC: miRNA loaded RISC; RNAi: RNA mediated interference; piRNA: Piwi interacting RNA; siRNA: small interfering RNA; ESR1: estrogen receptor 1; FOXL2: Forkhead box L2; DMRT1: double sex and mab3 related transcription factor 1; SOX9: SRY (sex determining region Y) box 9; NOBOX: newborn ovary homeobox gene; POF: premature ovarian failure; MRE: miRNA recognition element; NPM2: nucleoplasm 2; LH: luteinizing hormone; hCG: human chorionic gonadotropin; CTBP1: c terminal binding protein 1; FSH: follicle stimulating hormone; IGFBP3: insuling like growth factor binding protein 3; StAR: steroidogenic acute regulatory protein; BMPs: bone morphogenetic proteins; TGF- β1: transforming growth factor β; Smad4: Smad family member 4; LNA: locked nucleic acid; IFNGR1: interferon gamma receptor.

## Competing interests

The authors declare that they have no competing interests.

## Authors' contributions

JB: co-wrote the manuscript; JL: co-wrote, and designed structure of the manuscript. All authors read and approved the final manuscript.
